# Barriers and Motivators to Physical Activity Prior to Starting a Community-Based Walking Program

**DOI:** 10.3390/ijerph182010659

**Published:** 2021-10-12

**Authors:** Elizabeth A. Richards, Stephanie Woodcox

**Affiliations:** 1 School of Nursing, Purdue University, West Lafayette, IN 47907, USA; 2 Cooperative Extension, Purdue University, West Lafayette, IN 47907, USA; swoodcox@purdue.edu

**Keywords:** physical activity, intervention, social cognitive theory, walking, Cooperative Extension

## Abstract

Despite the clear benefits of an active lifestyle, most American adults fail to meet physical activity (PA) guidelines. Because of its safety and ease, walking is a promising population-level strategy to increase PA. There is a need to further understand why adults do and do not participate in walking. This study provides a broader understanding of barriers and motivators of walking prior to starting a walking program. Four years of baseline data from a community-based walking program were analyzed (n = 1491). Descriptive statistics summarized participant characteristics, barriers, reinforcements, and current PA. Chi-square tests were used to assess differences in the barrier and reinforcement responses between participant’s PA level and age categories. Open-ended responses were analyzed using thematic analysis. On average, participants were white (96%), middle-aged (52 ± 13 years old) females (92%). Poor weather and time were frequently reported barriers to walking. Open-ended responses (n = 141) identified additional barriers of lack of motivation (n = 37), joint issues (n = 29), fatigue (n = 24), safety or lack of environmental supports (n = 17), family or work demands (n = 15), and lacking a walking partner (n = 9). Good weather, health, and weight loss were frequently reported motivators. Additional motivators (n = 282) identified included stress relief and mental health (n = 82), social time (n = 70), dog care (n = 41), other health benefits (n = 38), connect with nature (n = 19), enjoyment (14), occupation (n = 11), and environmental and community supports (n = 6). Findings highlight the importance of understanding participant barriers and motivators for PA before starting a program. Future research should examine how reported barriers and motivators are related to program completion and adherence. Tailoring community-based programs to address specific barriers and motivators may enable more participants to effectively change and maintain PA.

## 1. Introduction

Physical inactivity is one of the main risk factors of preventable chronic diseases [1]. Alarmingly, the World Health Organization (WHO) [2] estimates that over one-quarter of adults across the globe are not meeting physical activity guidelines. Furthermore, physical inactivity is estimated to be associated with $117 billion dollars of annual health care costs in the United States alone [3]. Recognizing the global chronic disease burden related to physical inactivity, the WHO created a global target to reduce physical inactivity rates by 10% by 2025 and 15% by 2030 [2]. The WHO specifically mentions increasing walking behaviors as a population-level strategy to reduce rates of inactivity [2].

Promotion of walking has been recognized as an important health promotion strategy to combat inactivity [4]. Because walking is the most common form of physical activity for adults, it is considered an important strategy to not only increase, but maintain, physical activity behaviors [4]. Furthermore, walking can be a multi-purpose activity that not only results in increased physical activity but can also be an avenue for socialization [5] and improvement of mental health [6].

Effective community-wide intervention strategies are needed to facilitate an increase in and maintenance of walking behaviors across populations [7]. Existing studies have examined the barriers and motivators of walking in population subgroups. Lee and colleagues [8] reported that overweight and obese adults commonly reported environmental barriers of weather, poor lighting, and poor sidewalks as barriers to walking while access to recreational facilities were considered a motivator for walking behaviors. Specific to participation in a walking program, Perry [5] found that rural women considered family needs and chronic illness or injuries as barriers to program participation. Commonly reported motivators for program participation among these rural women included socialization, increased energy, and noticeable progress in walking behaviors [4]. Specific to older adults, common barriers to participating in a community walking program included illness and family commitments whereas motivators for program participation included socialization and anticipated health benefits [9]. During a primary care-based walking program, patients reported personal health, work and family commitments, and weather as barriers to participating in the walking program. Patients indicated that anticipated health improvements, social support, and enjoyment of walking were motivators to participate in a walking program [10].

Health behavior theories are important tools for both health promotion practitioners and researchers. These theories aid in the design, implementation, and evaluation of health promotion interventions by helping to identify addressable determinants of the targeted behavior. Social cognitive theory recognizes that perceived barriers and motivators to physical activity directly and indirectly influence behavior through self-efficacy [11]. It is theorized that perceiving positive outcomes such as enjoyment or health improvements facilitates behavior change and maintenance [12]. However, barriers to behavior change can lower self-efficacy which may result in relapse [13]. Importantly, many barriers to physical activity change are addressable and increasing attention to stated motivators for activity could further enhance program outcomes. Therefore, a broader understanding of barriers and motivators of walking behavior is important for community-based program development. The purpose of this study was to gain an understanding of barriers and motivators to walking among participants prior to starting Get WalkIN’, an email-based community-level walking program delivered through Cooperative Extension. Further, differences in reported barriers and motivators by participant subgroups of physical activity level and age were examined to inform nuances regarding the barriers and motivators for walking.

### Study Background

Get WalkIN’ is a twelve-week email-based program based on social cognitive theory [14] and delivered through one state’s Cooperative Extension System. Guided by social cognitive theory, the overarching goal of Get WalkIN’ is to increase participant self-efficacy by addressing commonly reported barriers while also promoting important motivators and reinforcements of walking behavior. To do so, email messages targeting constructs of self-efficacy, social support, self-monitoring, goal setting, barriers, motivators, relapse prevention are sent twice weekly for the first four weeks and then weekly for the following eight weeks [15].

Operating through a nationwide network of colleges, universities and land-grant educational institutions, the Cooperative Extension System, with support from the National Institute of Food and Agriculture, helps to bring educational programming and learning activities to communities and individuals. Through county-based Extension Educators, Cooperative Extension helps agriculture flourish; individuals and families maximize resources; enhance and grow life skills among adolescents; and contribute to improved health and well-being across the lifespan.

## 2. Materials and Methods

### 2.1. Study Design

This cross-sectional study utilized a quantitative and qualitative approach to examine participant-reported barriers and motivators to walking prior to starting an email-based walking program.

### 2.2. Participants and Recruitment

Over the course of four program years (June 2017–May 2021), county-based Extension Educators have recruited participants for Get WalkIN’. As part of the Get WalkIN’ toolkit [15], Extension Educators are given tailored recruitment materials which include social media messages, news releases, newspaper articles and flyers. Educators also recruited using pre-existing email listservs and during current Extension programs. Aside from being an adult, 18 years of age or older with access to email, there were no other limiting inclusion or exclusion criteria for participating in the program. Procedures were approved by the XXX University Committee on the Use of Human Research Subjects.

### 2.3. Measures

Prior to receiving the first Get WalkIN’ program email, participants received a survey link assessing baseline measures. Demographic variables included age, gender, race, highest level of education completed (less than high school, high school or GED, trade school, 2 or 4 year college, masters or professional degree, doctorate), and annual household income (<$40,000, $40–59,999, $60–69,999, $70–79,000, $80–89,999, and ≥$90,000). For ease of reporting purposes, categories of education and income were collapsed.

Based on prior studies identifying barriers to being active across the lifespan, barriers to walking were assessed with a shortened version of the CDC’s Barriers to Being Active Quiz [16]. Considering participant burden and the use of additional open-ended questions, four dichotomous items assessing poor weather (cold, hot, rain, snow), health, walking difficulty, and time-related barriers were chosen. Despite its public availability through the CDC, the Barriers to Being Active Quiz has not been widely tested. Studies which have used this scale have demonstrated high reliability (α = 0.92) [17]. Motivators to walking were assessed with four dichotomous items assessing good weather, weight loss, weight maintenance, and health motivators. It is important to highlight that personally modifiable barriers and motivators such as those that could be addressed through an email-based walking program, were chosen for participant assessment. Therefore, environmental barriers which could not be overcome by participants, such as lack of sidewalks, were not included in the pre-selected list. Further, rather than providing an exhaustive list of pre-selected barriers and motivator responses, participants were also asked to provide open-ended responses to identify additional barriers and motivators to walking.

Self-reported physical activity and walking was assessed using the International Physical Activity Questionnaire (IPAQ) Short-Form [18]. This self-report tool asks participants to report the average number of days they participate in walking, moderate, and vigorous activity in a typical week and the average duration in minutes per activity episode. To examine potential differences in reported barriers and motivators for walking, participants were categorized as high active (achieving at least 1500 MET minutes/week), moderately active (achieving 600–1499 MET minutes/week, or low active (achieving less than 600 MET minutes/week) based on IPAQ scoring protocols [18]. The IPAQ has demonstrated strong reliability (Spearman *p* ~0.80) and acceptable criterion validity in line with other self-report measures compared to objective measures (Spearman *p* ~0.35) [11].

### 2.4. Analysis

To be included in the analysis, participants needed to have complete physical activity data. Missing data on other variables ranged from 0.9% for gender to 15.5% missing for income. Descriptive statistics summarized participant characteristics, barriers, motivators, and physical activity and walking behavior. Chi-square tests were used to assess differences in the dichotomous barrier and reinforcement response between participant’s physical activity level (low active, moderate active, or high active) and age groups (≤34 years old, 35–54 years old; and ≥55 years old). The sample size for the qualitative data was much smaller and therefore cell sizes were too small to complete additional statistical analysis by physical activity and age. However, polar plots were created to visualize the thematic analysis of both barriers and motivators by physical activity and age categories. To account for differences in the number of participants per category, proportion of responses were visualized.

Open-ended responses were analyzed using thematic analysis [19]. Qualitative data were coded and categorized independently by two researchers. The first step of thematic analysis was familiarization [14,15]. Initially both coders read participant responses several times to familiarize themselves with the data. Second, a list of initial codes was created. A codebook was created to organize corresponding text that matched codes. Codes were collated into groups to allow for a condensed overview of the main ideas. Once consensus on codes was reached, codes were examined for patterns and commonalities to describe themes in reported barriers and motivators for walking. Social cognitive theory delineates that behavior change programs should intervene on psychosocial influences including internal barriers and motivators [20]. Therefore, themes were selected deductively based on prior research using social cognitive theory to examine barriers and motivators of walking [16,21,22]. Selected themes were then reviewed again with the qualitative data set to ensure important points were not left out or overstated. After this review, themes were named to provide succinct and understandable points for each topic.

## 3. Results

### 3.1. Participants

On average, participants were white (95%), middle-aged (52 ± 13 years old) females (91%) (see Table 1). A majority of participants had completed at least some college with 24.8% obtaining a master’s degree or higher. The physical activity levels of participants was evenly dispersed with 29.5% of participants classified as low active, 34.9% as moderately active, and 35.6% as high active (see Table 1).

### 3.2. Barriers to Walking

Most (91.5%) participants selected poor weather conditions such as heat, cold, rain or snow as barriers to walking. Half of participants (50.3%) also reported time as a barrier to walking. Barriers of personal health (6.5%) or having difficulty walking (8.3%) were less commonly reported (see Table 2).

Significant differences in barriers to walking were found for both physical activity level and age category. Low active participants and younger participants more often reported poor weather and time as barriers to walking. Low active and older participants more frequently reported difficulty with walking as a barrier. No significant differences were found with health as a reported barrier.

Seven themes were identified from the 141 responses to the open-ended question of additional barriers to walking (see Table 3). Lack of motivation (n = 37) was the most frequently reported theme followed by arthritis (n = 29) and fatigue (n = 27). In addition, participants reported concerns for safety or lack of environmental supports (n = 17) as barriers to walking. Family or work obligations (n = 15) were also identified as a barrier to walking. Less frequently reported was lack of enjoyment (n = 10) or lack of a walking partner (n = 9).

**Figure 1:**
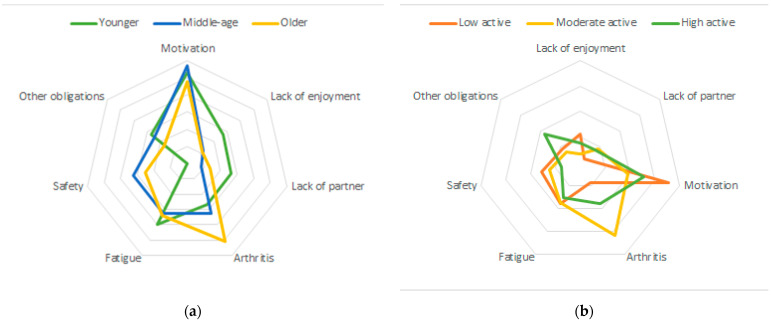
(**a**) Polar plot of thematic analysis of barriers by age category (younger n = 16; middle aged n = 52; older n = 73). (**b**) Polar plot of thematic analysis of barriers by physical activity category (low active n = 45, moderate active =57; high active =39).

In plotting the qualitative barrier themes by age category, it appears that lack of motivation is a prominent barrier across age groups. Arthritis appears more frequently in older participants and lack of a walking partner appears more frequently in younger participants (see Figure 1a). When plotting the qualitative barrier data by activity category, arthritis appears to be more of a barrier in moderately active participants whereas lack of motivation appears to be more of a barrier in low active participants (see Figure 1b).

### 3.3. Motivators to Walking

Heath promotion-related motivators (84.6%) to walking were frequently selected including weight loss (74.0%) and less often weight maintenance (32.8%). Pleasant weather was also frequently chosen as a motivator for walking (71.2%) (see Table 2).

Significant differences in motivators to walking were found for both physical activity and age category. High active and older participants were more likely to report health as a motivator for walking. Good weather was more likely to be a motivator for moderately active and younger participants. Weight loss, but not weight maintenance, was more frequently reported as a motivator in low active and middle-aged participants.

Eight themes of additional motivators were identified from the 282 responses to the open-ended question (see Table 3). Stress relief and mental health benefits were the most frequently mentioned motivator to walk (n = 82) followed by socialization opportunities (n = 70). Having a dog and participating in dog walking was another commonly reported motivator to walk (n = 41), followed by enjoyment of walking (n = 14) and connecting with nature (n = 19). Participants also mentioned many other health benefits obtained from walking as a motivator (n = 38) as well as having access to environmental and community supports (n = 7).

**Figure 2:**
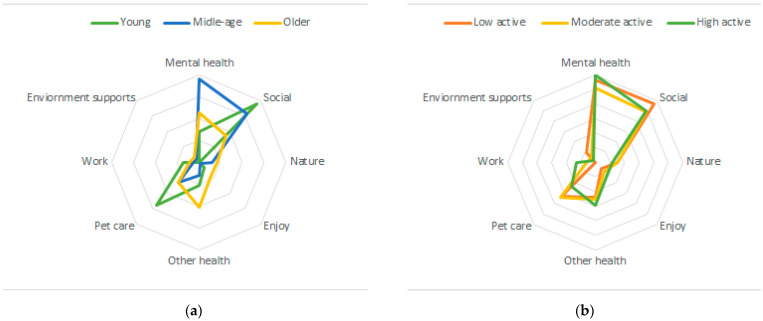
(**a**) Polar plot of thematic analysis of motivators by age category (younger n = 31; middle age n = 117; older n = 134). (**b**) Polar plot of thematic analysis of motivators by physical activity category (low active n = 68, moderate active =92; high active =122).

In plotting the qualitative motivator themes by age, psychosocial motivators appear more important than physical or environmental supports (see Figure 2a). Middle-aged participants more frequently reported themes of mental health motivators for walking whereas younger participants were more motivated by social reinforcements. Younger participants reported pet care as a frequent motivator whereas older participants reported mental health benefits and other health benefits of walking as fairly equal motivators. When plotting the qualitative motivator themes by physical activity level, reported motivators appear quite similar across levels of activity (see Figure 2b).

## 4. Discussion

The primary purpose of this study was to gain an understanding of participants’ reported barriers and motivators to walking prior to starting an email-based walking program. In addition, differences in reported barriers and motivators by physical activity level and age categories were examined. These findings could lead to a better understanding of barriers and motivators for walking, resulting in more tailored messaging for walking programs. Based on findings of this study, as discussed below, program materials for Get WalkIN’ were updated to further address participant-reported barriers and motivators.

Study findings highlight the importance of understanding potential barriers and motivators for walking when designing and implementing physical activity promotion programs. Of particular importance is addressing commonly reported barriers to walking early in health promotion programs. By addressing these barriers, participants may be more likely to continue to engage in the program and maintain increases gained in walking.

For example, having arthritis, and associated joint pain were commonly reported barriers to walking. However, routine walking can reduce joint pain associated with arthritis [6]. Specifically, walking and moderate intensity physical activity can improve pain management, physical functioning, and quality of life among those with arthritis [6]. E-mail materials for Get WalkIN’ were updated to specifically include a discussion of the therapeutic benefits of walking for arthritis and joint pain. Physical activity promotion programs should also provide clear messages that walking does not worsen the condition of arthritis, but rather is a therapeutic activity that can improve arthritis symptoms [23].

Feelings of fatigue and lack of energy were also frequently reported as barriers to walking. Therefore, Get WalkIN’ program emails were updated to specifically address fatigue-related barriers to walking and provide examples of how walking can combat feelings of fatigue. It is important to educate participants starting a walking routine that increasing physical activity levels can actually reduce feelings of fatigue and increase energy throughout the day [24]. Providing concrete facts about the role of exercise and fatigue could help participants recognize these benefits to moving more. For instance, one study found that inactive people with fatigue could increase energy up to 20% while reducing feelings of fatigue by up to 65% by engaging in regular activity [25]. In addition, using physical activity to combat feelings of fatigue can provide a cycle of benefits as studies have shown that feeling energized during physical activity can also promote future physical activity and facilitate exercise maintenance [26].

Based on participant responses of motivators for walking, walking programs should focus not only on the physical health benefits but also the mental health benefits of walking. Regular walking can reduce feelings of depression and anxiety [6] while also increasing feelings of being calm and happy [6]. Another powerful motivator for walking could be the importance of connecting with nature. Studies have found that when walking in nature, positive mood increases [27]. In fact, being active in nature can provide dual benefits. First, enjoying fresh air and connecting with the outdoors is independently associated with positive psychological well-being. The addition of physical activity while enjoying nature, referred to as green exercise, can promote further health benefits and facilitate an increase and maintenance of behavior change [27]. Based on these findings, Get WalkIN’ program emails were updated to expand the discussion of the mental health benefits of walking and included particular focus on mental health benefits from being active in nature.

Important differences in barriers and motivators for walking were found (both statistically and visually) between physical activity levels and age categories. These differences suggest it might be important to assess participant’s physical activity levels and demographics prior to program start and then subsequently provide tailored messaging based on the audience the program is serving. The least active participants and younger participants more frequently reported poor weather and time as barriers, suggesting that walking promotion programs might consider addressing these barriers early in the program. For example, strategies could be provided on how to add short walks or more steps in throughout the day rather than focusing on adding longer walks. In addition, providing ideas on alterative walking locations during bad weather could also help address barriers. When visually examining the qualitative barrier themes, lack of motivation appeared evenly across age groups but was more frequently reported among low active participants. Arthritis was more commonly reported as a barrier in older and in moderately active participants.

Walking programs which tailor the motivators based on populations the program is serving could also increase the adherence and maintenance of walking. For example, younger participants more often reported good weather as a motivator; middle-aged participants more frequently reported weight loss as motivation; and older participants more often reported health benefits as important motivators. Current (baseline) physical activity level is also important to consider when examining motivators for walking. High active participants more often reported weight maintenance as a motivator and low active participants more often reported weight loss as a motivator. In the visual examination of motivators by age and physical activity category, it appears that psychosocial motivators such as mental and social health benefits are more often reported that physical health or environmental supports. Focusing on these psychosocial motivators that are particularly important to the audience the program is serving could also increase adherence and maintenance behaviors.

These findings support the tenets of social cognitive theory and enhance our understanding of barriers and motivators to walking prior to starting a walking program. Specifically, our results suggest that focusing on both the mental health and the physical health benefits of walking are important motivators for behavior change. Further, future walking program should also consider addressing physical health barriers to walking such as pain and fatigue in addition to the more commonly reported barriers of time and lack of support.

Strengths of this study include the use of both quantitative and qualitative methods to explore participant-reported barriers and motivators to walking. By allowing participants to openly share barriers and motivators to physical activity, more nuanced and tailored promotion strategies may be developed. In addition, the established partnership of working with Cooperative Extension provided the ability have wide reach in participant recruitment. There are also study limitations worth noting. The participants recruited in this study were generally white women. While this is a common population served by Cooperative Extension, it does limit the generalizability of findings to a broader audience. In addition, these participants were already enrolled in this walking program when they reported perceived barriers and motivators. It is likely that perceptions of barriers and motivators may be different for adults who have yet to enroll in a physical activity promotion program. Furthermore, it is important to consider that this program is offered in a distance format, via email. Adults considering joining an in-person walking program or walking group may report different barriers and motivators to their physical activity.

## 5. Conclusions

Our results add to a growing body of literature that highlights the importance of understanding participant barriers and motivators for physical activity before starting a walking program. Evidence from this study could lead to more specific messages and intervention strategies based on physical activity and age characteristics of program participants. Results of this study suggest that community-based walking promotion programs should address barriers of time, arthritis, and motivation while highlighting motivators beyond physical health to also include social and mental health. Tailoring community-based programs to address specific barriers and motivators may enable more participants to effectively change and maintain increases gained in physical activity. Future research should examine how reported barriers and motivators are related to program completion and adherence.

## Figures and Tables

**Table 1:** Participant characteristics ^1^ (n = 1491).

	Mean	Standard Deviation
Age	52.1	13.4
	N	%
Gender		
Male	122	8.2
Female	1356	90.9
Race		
White	1416	95.0
Non-White	58	3.9
Education		
High school	214	14.4
Some college	282	18.9
2–4 year college	608	40.8
Master’s or higher	369	24.8
Household income		
<$40,000	248	16.6
$40,000–$69,999	372	25.0
$70,000–$89,999	243	16.3
≥$90,000	396	26.6
Physical activity level		
Low active	440	29.5
Moderately active	520	34.9
High active	531	35.6

^1^ Not all categories add up to 100% due to some missing data.

**Table 2:** Participant-reported barriers and motivators to walking (n = 1491).

	Total (n = 1491)	Low Active (n = 459)	Moderate Active (n = 509)	High Active (n = 522)	Young (n = 158)	Middle Aged (n = 602)	Older Aged (n = 577)
Barriers (reported yes)	N (%)	N (%)	N (%)	N (%)	N (%)	N (%)	N (%)
Poor weather **^+^	986 (66.1)	336 (73.2)	338 (66.3)	312 (59.8)	116 (73.4)	405 (67.2)	418 (63.1)
Time *^++^	746 (50.3)	252 (55.0)	260 (51.0)	234 (45.4)	102 (64.6)	363 (60.6)	243 (36.8)
Difficulty walking **^++^	123 (8.3)	64 (14.0)	38 (7.5)	21 (4.1)	13 (8.2)	32 (5.3)	74 (11.2)
Health **	97 (6.5)	48 (10.5)	26 (5.1)	23 (4.5)	11 (7.0)	37 (6.2)	46 (7.0)
Motivators (reported yes)							
Health *^++^	1260 (84.6)	383 (83.4)	418(82.1)	459 (87.9)	121 (76.6)	501 (83.2)	577 (87.0)
Good weather **^++^	1061 (71.2)	315 (68.6)	389 (76.4)	357 (68.4)	130 (82.3)	428 (71.1)	457 (68.9)
Weight loss **^++^	1102 (74.0)	361 (78.7)	382 (75.1)	359 (68.8)	113 (71.5)	475 (78.9)	462 (69.7)
Weight maintenance **	489 (32.8)	98 (21.4)	171 (33.6)	220 (42.2)	47 (29.8)	198 (32.9)	225 (33.9)

Significantly different by physical activity category * *p* < 0.05; ** *p* < 0.01. Significantly different by age category + *p* < 0.05 ++ *p* < 0.01.

**Table 3:** Thematic analysis of open-ended responses of barriers and motivators.

	N	%	Example Quotes
Barriers (n = 141)			
Lack of motivation	37	26.2	“I have no ambition to walk” “I’m just plain lazy” “I am really not motivated to walk”
Arthritis	29	20.6	“My knees hurt me” “I have hip pain” “I have arthritis in my ankle”
Fatigue	27	17.0	“I am too tired after work” “I don’t have enough energy, I need to rest” “At the end of the day I am exhausted”
Safety	17	12.1	“I am scared of being alone” “I don’t live in a safe area” “I don’t like to walk by myself when it is dark” “There are no sidewalks or paths easily available”
Family or work obligations	15	10.6	“I have too many family commitments” “I am always being a caretaker” “I need to be on my computer for work”
Lack of enjoyment	10	7.1	“I would rather do other things” “I don’t like to walk” “I have other interests”
Lack of a partner	9	6.4	“I don’t have a walking buddy” “My husband doesn’t want to walk with me”
Motivators (n = 282)			
Stress relief and mental health	82	29.1	“I walk because it helps with my stress” “To de-stress and get out of the office” “Walking helps me gather my thoughts” “…to lighten my mood”
Socialization	70	24.8	“Walking gives me time with my spouse” “I like to walk with my family” “It gives me quality time with my son” “I get to talk with my friends”
Dog walks	41	14.5	“I have a new puppy” “My dogs need to walk” “I like to spend time with my dog”
Other health benefits	38	13.5	“Walking helps improve my sleep” “It helps my joints feel better” “I want to get off blood pressure medicines”
Connect with nature	19	6.7	“I enjoying being in nature and seeing wildlife” “I get relaxation from looking at the trees and the sky” “I feel connected to being outside”
Enjoyment	14	5.0	“I enjoy seeing my neighborhood” “I enjoy walking, it makes me happy”
House work or occupation	11	3.9	“Parking farther at work” “When I have heavy house chores” “Gardening and yard work”
Environmental or community supports	7	2.5	“Good sidewalks” “Access to places to walk to”

## Data Availability

The data presented in this study may be available on request from the corresponding author. The data are not publicly available due to continued data collection and analysis.
